# Epigenetics and Beyond: Targeting Histone Methylation to Treat Type 2 Diabetes Mellitus

**DOI:** 10.3389/fphar.2021.807413

**Published:** 2022-01-11

**Authors:** Yang Yang, Ying Luan, Qi Feng, Xing Chen, Bo Qin, Kai-Di Ren, Yi Luan

**Affiliations:** ^1^ Department of Translational Medicine Center, the First Affiliated Hospital of Zhengzhou University, Zhengzhou, China; ^2^ Department of Physiology and Neurobiology, School of Basic Medical Sciences, Zhengzhou University, Zhengzhou, China; ^3^ Research Institute of Nephrology, Zhengzhou University, Zhengzhou, China; ^4^ Department of Pharmacy, the First Affiliated Hospital of Zhengzhou University, Zhengzhou, China; ^5^ Henan Key Laboratory of Precision Clinical Pharmacy, Zhengzhou University, Zhengzhou, China

**Keywords:** type 2 diabetes mellitus, histone methylation, histone methyltransferases, histone demethylases, epigenetic-based therapies

## Abstract

Diabetes mellitus is a global public health challenge with high morbidity. Type 2 diabetes mellitus (T2DM) accounts for 90% of the global prevalence of diabetes. T2DM is featured by a combination of defective insulin secretion by pancreatic β-cells and the inability of insulin-sensitive tissues to respond appropriately to insulin. However, the pathogenesis of this disease is complicated by genetic and environmental factors, which needs further study. Numerous studies have demonstrated an epigenetic influence on the course of this disease *via* altering the expression of downstream diabetes-related proteins. Further studies in the field of epigenetics can help to elucidate the mechanisms and identify appropriate treatments. Histone methylation is defined as a common histone mark by adding a methyl group (-CH3) onto a lysine or arginine residue, which can alter the expression of downstream proteins and affect cellular processes. Thus, in tthis study will discuss types and functions of histone methylation and its role in T2DM wilsed. We will review the involvement of histone methyltransferases and histone demethylases in the progression of T2DM and analyze epigenetic-based therapies. We will also discuss the potential application of histone methylation modification as targets for the treatment of T2DM.

## Introduction

Diabetes mellitus, a global public health challenge with rapidly increasing morbidity rate, causes a high epidemiological and economic burden on health systems worldwide (Zimmet et al., 2016). This disease serves as a high-prevalence epidemic that currently affects approximately 316 million people, which is estimated to reach 470 million by the year 2035 (Reed et al., 2021). Among the types of diabetes, type 2 diabetes mellitus (T2DM) is a common form, which accounts for nearly 90% of the global prevalence of diabetes (Dendup et al., 2018). Type 2 diabetes is a complicated, chronic, and multi-factor disease, featured by prolonged high glucose levels, altered insulin sensitivity, pancreatic beta cell dysfunction, and alterations in oxidative and inflammation-related gene expression (Venables and Jeukendrup, 2009). Intuitively, T2DM is induced either by insulin resistance (IR) from insulin-responsive cells and tissues or pancreatic β‐cell dysfunction, leading to inadequate secretion of insulin (Galicia-Garcia et al., 2020). Mechanically, T2DM is due to a combination of genetic and environmental factors. Genetic factors include heritable polymorphisms and mutations in genes that are responsible for regulating insulin sensitivity. Environmental factors include unhealthy diet, old age, and sedentary lifestyle, playing essential roles in T2DM progression (Lee et al., 2000). As depicted by a follow-up epidemiology of diabetes intervention and complication study, patients with diabetes who underwent standard insulin therapy show persistent slight inflammation and progressive vascular complications despite the intensified therapy afterward, indicating a potential metabolic memory signature prompted by hyperglycemia (Ryk et al., 2020). However, the pathogenesis of this disease is complicated by genetic and environmental factors, which need further study. Numerous studies have demonstrated an epigenetic influence on the course of this disease. Further research in the field of epigenetics can help to elucidate the mechanisms and identify appropriate treatments.

Epigenetics refers to somatic heritable genetic traits caused by changes in chromatin structure without changing the DNA sequence, including DNA methylation, histone modifications, noncoding RNA (ncRNAs) regulation, and chromosomal remodeling (Ordog et al., 2012). Among the epigenetic modifications, DNA methylation is a specific postsynthetic, enzymatic modification of DNA base. In addition, histone modification is a covalent post-translational modification (PTM) to histone proteins, whereas ncRNA modification occurs at the post-translational and post-transcriptional levels (Cedar and Bergman, 2009). A nucleosome, which is known as the basic unit of chromatin, is composed of 146 bp DNA sequences intertwining a core histone octamer, including two copies of H2A, H2B, H3, and H4 (Mariño-Ramírez et al., 2005). The nucleosome further assembles into a spiral fiber with six nucleosomes per circle with the assistance of other proteins, such as histone H1. The N-terminal of histones can be covalently modified by various types of PTMs, including acetylation, methylation, phosphorylation, and ubiquitination (Ramazi et al., 2020).

Based on the transcriptional status, chromosome exists in two different functional states in cells. It is either in a highly folded condensed structure that is, unavailable for transcription (heterochromatin) or in an unfolded, uncondensed structure that is accessible for transcriptional factors to initiate gene transcription (euchromatin) (Pederson and Robbins, 1972). Despite early observations, their functional explorations are just at the beginning stage. Nuclear histone acetyltransferase (HAT) is initially identified as a homolog of the yeast transcriptional coactivator *Gcn5p*, which correlates with the findings of a previous study, that is, histone acetylation is related to gene transcriptional activation (Verdone et al., 2006). These observations induce in-depth research of histone acetylation function in gene transcription modulation. Therefore, biochemical and genetic analyses show the importance of histone acetylation in transcriptional regulation. As shown in previous studies, several factors can modulate the status of acetylation, including HATs (e.g., Gcn5, p300/CBP, PCAF, TAF250, and the p160 family) and histone deacetylases (HDACs, e.g., Sin3 and NCoR/SMRT) (Zhang and Reinberg, 2001). All these studies have confirmed the regulation of the dynamic structure of chromatin by histone acetylation. Lysine acetylation has been implicated in mediating immunological and metabolic pathways; therefore, it maintains energy homeostasis *via* controlling the expression of downstream proteins (Iyer et al., 2012). Lysine acetylation can also affect the expression of a majority of the metabolic enzymes involved in glycolysis, tricarboxylic acid (TCA) and urea cycles, and fatty acid and glycogen metabolism in the liver (Kosanam et al., 2014). For example, the enhanced deacetylation activity of SIRT1 is observed in caloric restriction and fasting-mediated fatty acid oxidation, which maintains glucose homeostasis, accompanied by PGC-1α and PPARα activation (Soyal et al., 2006). SIRT1 activation also suppresses the expression-targeted genes of SREBP regulatory elements and improves metabolic status in diet-induced and genetically obese mice (Ding et al., 2017). These results suggest the modulatory effects of deacetylases and the acetylation levels on metabolism. The inhibitors of HDACs can also mediate the development, proliferation, and differentiation of β-cells in diabetic animal models and IR (Khan and Jena, 2014).

Apart from histone acetylation, critical achievements have been made in the studies of other histone modifications, such as phosphorylation and histone methylation (Bui et al., 2010). These modifications are mutually affected, and pre-formed modifications can modulate subsequent histone modifications. Collectively, these modifications serve as marks for recruiting other proteins to control diverse chromatin functions, including gene expression, DNA replication, and chromosomal segregation (Zhang and Reinberg, 2001).

Accumulating studies have shown that histone modifications interact and influence each other because the decoding of a specific post-translational modification (PTM) at a single-nucleosome level is difficult (Rothbart and Strahl, 2014). At present, advances in high-throughput technology facilitate the protein modification identification on a large scale. Notably, recent studies have recognized histone methylation as the most flourishing field of epigenetics and most stable type of PTM (Ishii et al., 2021).

Previous studies have mostly focused on the effects of epigenetic on some of the major physiological and pathological processes, such as embryonic development, aging, and cancer (Khan et al., 2016). However, considerable attention has been paid to other fields recently, such as inflammation, obesity, cardiovascular diseases, neurodegenerative diseases, and immune diseases (Khan et al., 2016; Jasiulionis, 2018). Considering that the epigenetic modifications are susceptible to external and internal factors and are capable to modulate gene expression, epigenetic is regarded as the unknown and potential critical mechanism of several diseases. Epigenetic modifications are inheritable during cell division, leading to stable inheritance of acquired phenotypes; therefore, epigenetics can serve as a new framework for the exploration of etiological factors in environment-associated diseases, particularly diabetes.

## Histone Methylation

**FIGURE 1:**
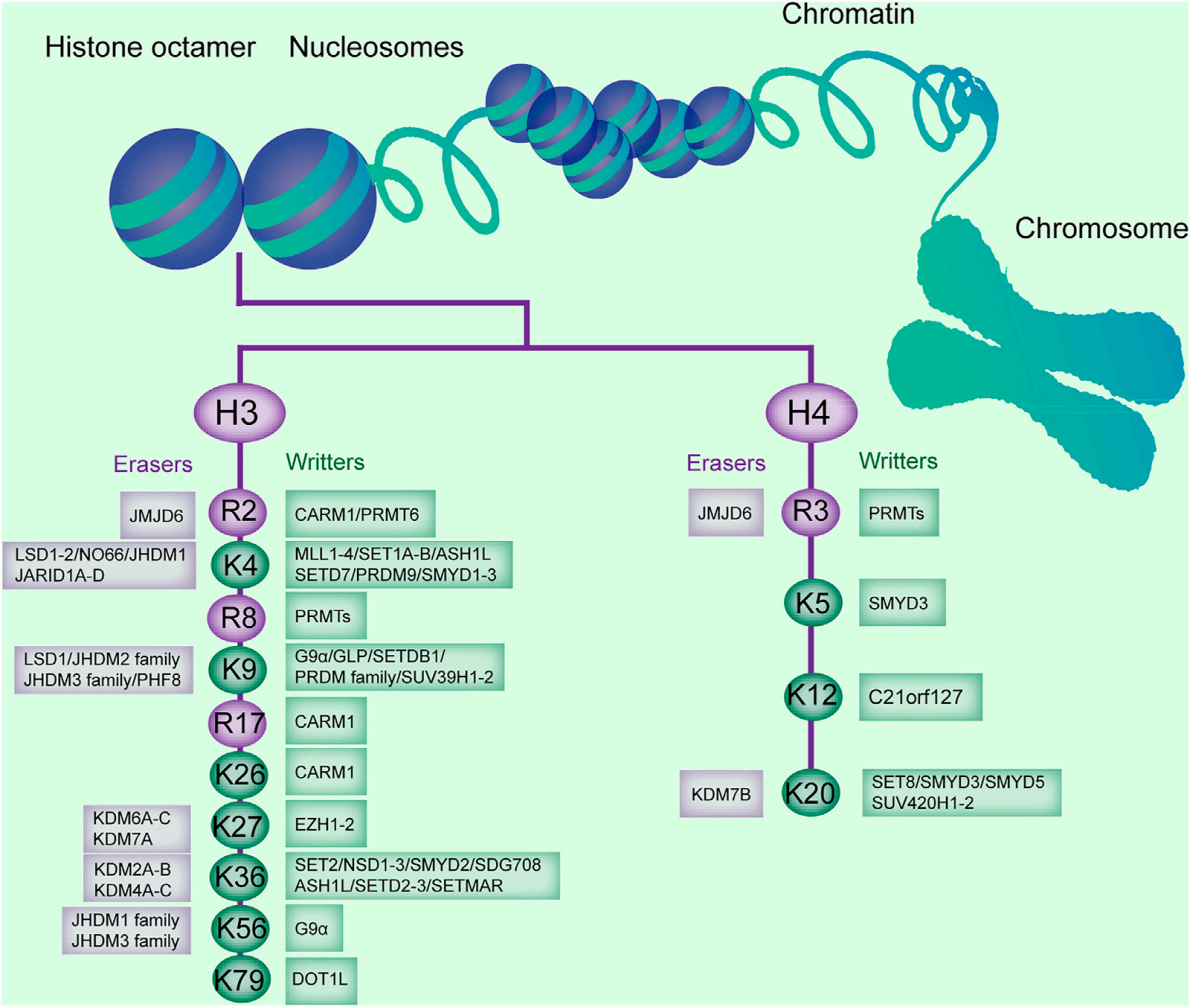
Histone methylation sites on H3 and H4, and the enzymes catalyzing or removing histone methylation. The existing methylation sites in histone H3 and H4 and the histone methyltransferases (writers) and demethylases (erasers) responsible for these modification sites are listed.

Histone methylation is a common histone mark by adding a methyl group (−CH_3_) onto a particular lysine or arginine residue (Greer and Shi, 2012). The methylation on the lysine residues can be mono-(me), di-(me2), or tri-(me3) on the ε-amino group, whereas for arginine, it can be mono-(me) or di-(me2s) symmetrically or asymmetrically (me2a) (Kim et al., 2019). Histone methylation can be added by histone methyltransferase (HMTs), catalyzing the transfer of a methyl group from S-adenosylmethionine to their targeted residues (Yang et al., 2021). Typically, HMTs are composed of three families: the SET-domain containing enzymes, Dot1-like proteins, and arginine N-methyltransferase enzymes (PRMTs, Figure 1; Table 1) (Michalak and Visvader, 2016). The former two families primarily act on lysines (KMTs) and share a conserved catalytic SET domain that is, originally identified in the Drosophila Su [var] 3-9, Enhancer of zeste, and Trithorax proteins, and the latter primarily acts on arginines (Bhaumik et al., 2007; Park and Han, 2019). HMTs not only methylate histones that make up for chromatin, but also free histones and even non-histone proteins (Gong and Miller, 2019).

**TABLE 1:** Known histone methylation sites and proposed functions.

Histone	Site	Histone-modifying enzymes	Proposed function	Alteration in metabolism
H1	Lys26	EZH2	transcriptional silencing	
H2A	Arg3	PRMT1/6, PRMT5/7	transcriptional activation, transcriptional repression	
H3	Arg2	PRMT5, PRMT6	transcriptional repression	
	Arg8	PRMT5, PRMT2/6	transcriptional activation, transcriptional repression	
	Arg17	CARM1	transcriptional activation	correlates with insulin gene expression and insulin secretion stimulated by glucose
	Arg26	CARM1	transcriptional activation	
	Arg42	CARM1	transcriptional activation	
	Lys4	Set1 (S. cerevisiae)	permissive euchromatin (di-Me)	enhanced in response to hyperglycemia, enhanced in uninephrectomized db/db mice
		Set 7/9 (vertebrates)	transcriptional activation (tri-Me)	
		MLL, ALL-1	transcriptional activation	
		Ash1 (D. melanogaster)	transcriptional activation	
	Lys9	Suv39h, Clr4	transcriptional silencing (tri-Me)	reduced in VSMCs stimulated with high glucose and diabetic mice model
		G9a	transcriptional repression genomic imprinting	
		SETDB1	transcriptional repression (tri-Me)	
		Dim-5, Kryptonite	DNA methylation (tri-Me)	
		Ash1	transcriptional activation	
	Lys27	Ezh2	transcriptional silencing	reduced in T2DM and T1DM.
			X inactivation (tri-Me)	
		G9a	transcriptional silencing	
	Lys36	Set2	transcriptional activation (elongation)	higher H3K36me3 in db/db mice
	Lys79	Dot1	euchromatin	associated with glucose-stimulated insulin secretion
			transcriptional activation (elongation)	
			checkpoint response	
H4	Arg3	PRMT1/6	transcriptional activation	elevated in response to glucose dysregulation and HFD
		PRMT5/7	transcriptional repression	
	Lys20	PR-Set7	transcriptional silencing (mono-Me)	
		Suv4-20 h	heterochromatin (tri-Me)	
		Ash1 (D. melanogaster)	transcriptional activation	
		Set9 (S. pombe)	checkpoint response	
	Lys59	unknown	transcriptional silencing	

Conversely, histone demethylases (HDMs) promote the removal of a methyl group from lysines or arginines. Lysine demethylases (KDMs) are classified into two families: the FAD-dependent amine oxidases and Fe (II)- and α-ketoglutarate-dependent jumonji C (JmjC)-domain containing iron-dependent dioxygenases (JMJD, Figure 1) (Arifuzzaman et al., 2020).

Lysine-specific demethylase (LSD) is composed of two members, LSD1 and LSD2, and demethylase mono- and dimethylated H3K4 and H3K9 (Kim et al., 2018). JmjC domain-containing HDMs are divided into several subgroups, including the JARID/KDM5, JMJD1/JHDM2/KDM3, JMJD2/KDM4, JMJD3/KDM6, JHDM1/FBX/KDM2, and JmjC domain-only group, based on the substrate specificity for H3K4, H3K9, H3K27, or H3K36 (Dong et al., 2020). The JMJD2 or KDM4 family, containing JMJD2A (KDM4A), JMJD2B (KDM4B), and JMJD2C (KDM4C), is responsible for demethylation of di- and trimethylated H3K9 and H3K36 (H3K9me2/me3 and H3K36me2/me3, Figure 1; Table 1) (Kim et al., 2018). Arginine demethylases remain less represented.

Histone modifications at particular loci are associated with the transcriptional status of the downstream genes (Ellis et al., 2009). Hyperacetylation of H3 and H4 is associated with the activation of gene transcription; conversely, hypoacetylation correlates with the repression of gene transcription (Schnekenburger et al., 2007). Histone methylation can be an activator and repressor of downstream gene transcription. For example, H3K9me2 is commonly believed as repressive heterochromatin. H3K36 methylation has been shown to associate with transcriptional activation (Matsuda et al., 2015). Lysine methylation is observed in transcriptional activation (H3K4, K36, K79) and repression (H3K9, K27, H4K20) (Table 1) (Völkel and Angrand, 2007). In addition, the extent of methylation affects the status of gene transcription. H4K20 monomethylation (H4K20me1) is associated with active gene transcription, whereas trimethylation on H4K20 (H4K20me3) correlates with silenced gene transcription and compacted heterochromatin (Li et al., 2011). The status of gene transcription is also modulated by the loci of the methylation with regard to the DNA sequence. For example, H3K9me3 at the promoter is associated with gene repression, and that at the gene body is correlated with gene activation (Nagy et al., 2017). The influence of histone modification on gene transcription is determined through recognition of other binding motifs. Several chromatin-modifying enzymes are involved in histone methylation on recruitment to specific target gene loci (Bannister and Kouzarides, 2011). For example, the Tudor domains (e.g., 53BP1 and JMJD2A/KDM4A) are found in the methyl-lysine-binding module of histone methylation enzymes (Mallette et al., 2012).

## Histone Methylation in Type 2 Diabetes

Type 2 diabetes results from the combined interaction between genetic and environmental factors (Ahlqvist et al., 2011). However, the genetic factors identified at present only account for a small percentage of the observed disease. The remaining heritability could be possibly explained by rare variants, including gene-environment interactions and epigenetics. Aberrant histone modifications prelude the development of various diseases, such as IR (Maude et al., 2021). Several studies have focused on the relation of histone modification and T2DM.

**FIGURE 2:**
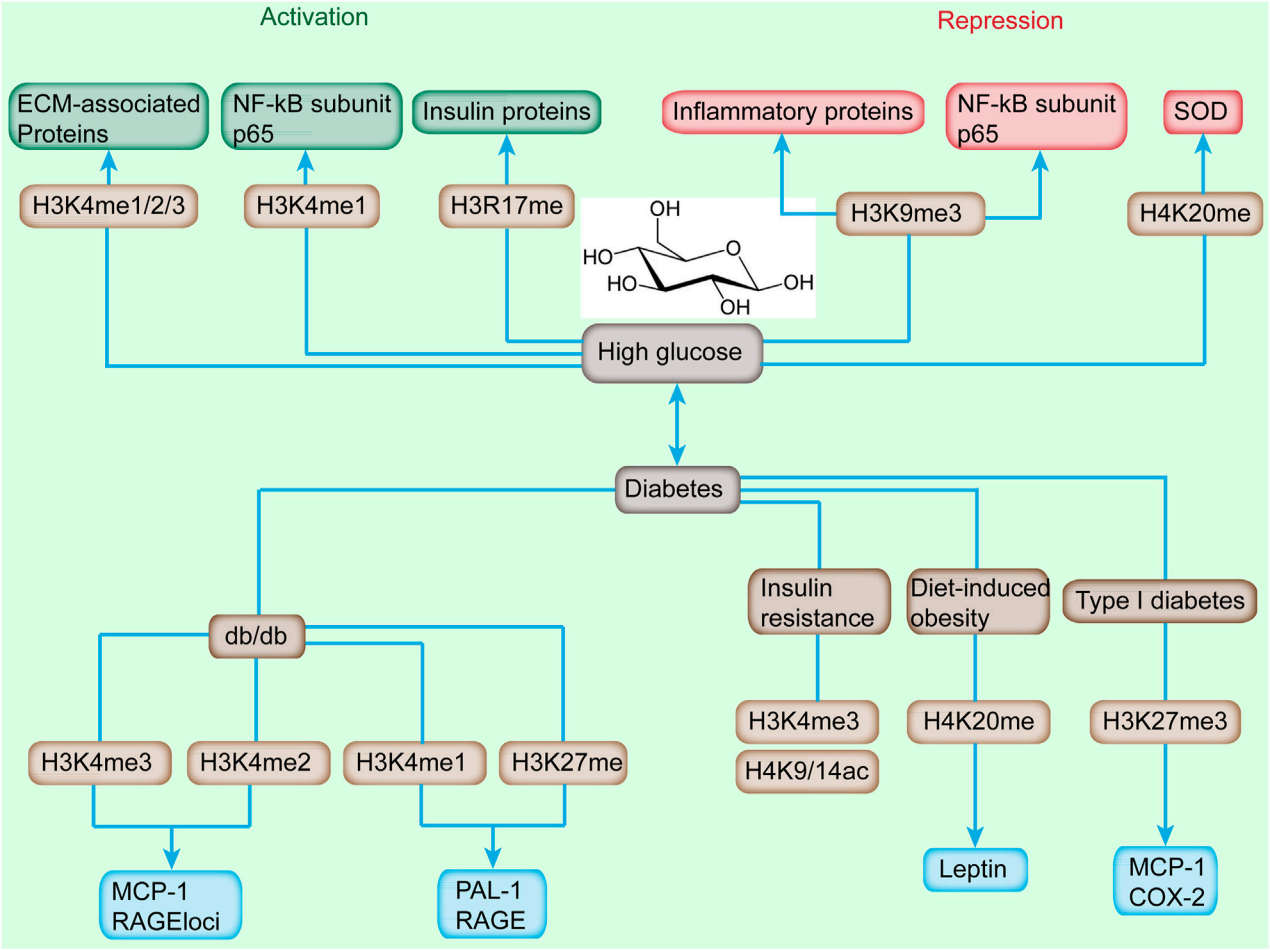
Alterations of histone methylation and downstream-targeted gene induced by high glucose and diabetes. High glucose induces the increased level of H3K4me1/2/3 and H3R17me and reduces the landscape of H3K9me3 and H4K20me, which cause the activation or repression of downstream genes. Diabetes, featured by sustained high glucose, is accompanied with increased H3K4me2/3, and decreased level of H3K4me1, H3K27me, H3K4me3, H4K9/14ac, H4K20me, and H3K27me3 and upregulation or downregulation of targeted genes.

Insulin resistance is a common characteristic found in many metabolic defects, including high fasting glucose, high triglycerides, low high-density lipoprotein cholesterol, and hypertension (Roberts et al., 2013). Sustained IR could induce T2DM, which is the pathology of T2DM for a long time. Broad examinations of histone modifications in IR have been performed, including hepatic IR, T2DM, and obesity (Castellano-Castillo et al., 2019). For example, the progression of T2DM is accompanied by an increased level of H3K4me1 and H3K9me2 and a reduced level of H3K9ac and H3K23ac (Figure 2) (Tu et al., 2015). Global proteomic analysis revealed 15 histone modifications differentially abundant in high-fat diet (HFD)-induced mice (Maude et al., 2021). HDAC8 was associated with the promotion of IR in NAFLD-associated hepatocellular carcinoma (HCC). About 5,000 regions of H3K27ac enrichment were found to be significantly different in HFD-induced glucose-tolerant mice. Many genes are reported to have altered expression and contribute to the pathogenesis of T2DM. Increased expression and decreased methylation of *CDKN1A* and *PDE7B* genes in T2DM were reported to result in impaired glucose-stimulated insulin release (Ishikawa et al., 2015). The high H3K4me3 level of the *Fxyd3* gene negatively modulates glucose competence of insulin-secreting cells in mice (Vallois et al., 2014). ER stress induced the increased level of H3K4me1 at the inflammatory gene *MCP-1* promoter in accordance with the enhanced expression of *MCP-1* by SET7/9 upregulation in db/db mice (Chen et al., 2014). These observation provides evidence of diabetes associated epigenetic modifications and associated impaired insulin release.

Initial studies analyzed DNA methylation of candidate genes for T2DM such as *INS* (encoding insulin), *PDX1*, *PPARGC1A* (encoding PGC1α), and *GLP1R* (encoding the GLP-1 receptor) in human pancreatic islets from donors with T2DM and non-diabetic controls (Ling and Rönn, 2019). Islets from T2DM donors were found to have increased DNA methylation and decreased expression of these key genes, which were associated with impaired insulin secretion. Notably, genome-wide histone modifications have so far only been analyzed in pancreatic islets of non-diabetic subjects, whereas other studies performed in blood cells have included subjects with T2DM. In addition, histone modifications have been analyzed in monocytes cultured in normal and high glucose. The same applies for analysis of the chromatin structure, for example, by ATAC-seq, where mainly samples from non-diabetic people have been used. Therefore, there is a large need for further epigenome-wide studies in tissues from subjects with T2DM.

### Active Chromatin Marks

H3K4me1/2/3, H3K36me2/3, and H3K79me2 are correlated with transcriptional activation (Zheng et al., 2021). A genome-wide study of these modifications revealed that these modifications played critical roles in the specific promoters and enhancers of the islet and pathogenesis of diabetes (Backe et al., 2019).

Recently, H3K4me1/2/3 has been implicated in the dysregulation of critical genes involved in the progression of diabetic nephropathy (DN) (Figure 2) (Kato and Natarajan, 2014). An increased level of H3K4me landscape at extracellular matrix-associated gene promoters was observed in rat mesangial cells (RMC) upon high-glucose treatment (Sun et al., 2010). In transient hyperglycemia endothelial cells, the level of H3K4me1 at the proximal promoter of p65 was increased accompanied with the sustained activation of NF-κB subunit p65 (Wegner et al., 2014). Meanwhile, the enhanced H3K4me1 level at the p65 promoter was induced by hyperglycemic exposure in aortic endothelial cells isolated from non-diabetic mice (Rajasekar et al., 2015). ChIP coupled with DNA microarray (ChIP-on-chip) assay depicted that high-glucose stimulation altered the level of H3K4me2 in cultured human monocytes (Sun et al., 2014). Moreover, the H3K4me2 level was enhanced in uninephrectomized db/db mice.

The methylation of histones correlated with the progression of T2DM. For example, treatment with MCP-1/CCL2 antagonist relieved DN histological damage and H3K4me2 methylation status in uninephrectomized db/db mice (Figure 2) (Sun et al., 2014). In type 2 diabetic db/db mice, the H3K4me1 level was enhanced, which was consistent with enhanced RNA polymerase II recruitment at the promoter region of PAI-1 and RAGE (receptor for advanced glycation end products) compared with db/+ mice; however, H3K4me2/3 showed no evident difference (Sun et al., 2014). In addition, ER stress induced the increased level of H3K4me1 at the *MCP-1* promoter in accordance with the enhanced expression of inflammatory gene *MCP-1* by SET7/9 upregulation in db/db mice (Shao et al., 2021). H3K4me3 enhancement was observed in proinflammatory genes (*MCP-1* and *TNF-*α), profibrotic genes (*TGF-β1* and *collagen III*), and histone-modifying enzyme (*SET1* and *BRG1*) in an ischemia-reperfusion injury animal model (Naito et al., 2009). In IR state, H3K4me3 and H3K9/14ac were reduced in adipose tissue (Figure 2) (Wróblewski et al., 2021). The enhancement of the H3K4me3 level promoted PEPCK expression, which might contribute to hyperglycemia and anorexia in mandarin fish fed on carbohydrate-rich diets (You et al., 2020). The high H3K4me3 level of the *Fxyd3* gene negatively modulates glucose competence of insulin-secreting cells in mice (Figure 2) (You et al., 2020). Moreover, transcription factor 19 regulates gluconeogenesis through mediating H3K4me3 and modulating the expression of glucose-6-phosphatase and fructose-1,6 bisphosphatase (Sen et al., 2017). Diet-induced obesity in mice depicted the significantly reduced level of acetylated H3, H4, and methylated H4K4 (Milagro et al., 2013).

H3K4me2 demethylation is mediated by a FAD-dependent demethylase, LSD1, which is enhanced in adipose tissue of HFD-induced mice (Hino et al., 2012). In addition, the genes related to energy expenditure are directly repressed by LSD1. Thus, the inhibition of LSD1 elevates the entire H3K4 methylation and reduces the body weight of HFD-induced mice (Hino et al., 2012). A HMT, MLL3, could induce H3K4 methylation (Bosgana et al., 2020). The mutations of MLL3 at the catalytic SET domain change the level of a series of metabolic genes such as *Rbp4*, which is involved in insulin sensitivity. These studies demonstrate that manipulating the global level of H3K4 methylation could affect adiposity and insulin sensitivity (Jufvas et al., 2013). This finding is further verified by studying the positive correlation of the expression level of H3K4me3 and PPARγ during adipogenesis (Wang et al., 2013). Moreover, highly demethylated H3K4 is observed at the insulin reporter, indicating the involvement of histone methylation in insulin promoter modulation (Žumer et al., 2012).

The methylation on histone H3 Arg17 and Arg26 has been considered as transcriptional activation (Selvi et al., 2010). H3R17 methylation mediated by PRMT4 correlates with insulin gene expression and insulin secretion stimulated by glucose in pancreatic β cells (Ma et al., 2001). Another activation mark, H3K36me3, was reported to be correlated with transcriptional elongation (Sun et al., 2020).

Different from other methylation sites at the histone tails, the methylation of H3K79 is on the globular domain of the histone (Mushtaq et al., 2021). Its methylation is mediated by the methyltransferase, which is a disruptor of telomeric silencing proteins DOT1/DOT1L (Farooq et al., 2016). H3K79 methylation is involved in mediating the cell cycle, embryonic development, DNA damage response, and hematopoiesis (Farooq et al., 2016). The ring-finger ubiquitin ligase complex components, namely, Rnf20 and Rnf40, are a prerequisite for histone modifications such as H3K4 and H3K79 methylation (Jääskeläinen et al., 2012). Rnf complex depletion affects the expression of β-cell genes, including *Glut2*, *MafA*, and *Ucp2*, contributing to the reduced glucose-stimulated insulin secretion.

### Repressive Chromatin Marks

Histone methylation has several repressive chromatin marks, which could affect the progression of T2DM. H3K9me2/3, H3K27me3, and H4K20me3 are generally considered as gene silencers (Li et al., 2019). These histone methylations are involved in metabolic memory leading to long-term alterations in diabetes (Sun et al., 2014). The decreased H3K9me3 level at the promoter of inflammatory genes (*IL-6*, *MCSF*, and *MCP-1*) was induced, accompanied with increased expression in response to high glucose in normal human vascular smooth muscle cells (VSMCs) (Villeneuve et al., 2008). Similar methylation alterations were observed in VSMCs of a diabetic mice model (Villeneuve et al., 2010). In addition, the stimulation of TNF-α further exacerbated the decrease of H3K9me3 along with increased expression of inflammatory genes in VSMCs of db/db mice (Sun et al., 2014). In other RMC models, H3K9me2/3 was reduced, accompanied with induced upregulation of *Col1α1*, *PAI-1*, and *CTGF* genes in response to TGF-β and high glucose (Sun et al., 2017). Moreover, transient stimulation with high glucose triggered sustained reduction of the H3K9me2 and H3K9me3 levels at the p65 promoter, even after removing high glucose, indicating that the epigenetic alterations were remarkable metabolic changes (Wei et al., 2020). Furthermore, the dynamic epigenetic modification of H3K9me2 was observed in THP-1 cells when high glucose and monocytes were isolated from patients with diabetes (Miao et al., 2007). The expression of p66Shc was modulated by a complicated network, including the methyltransferase SUV39H1, demethylase JMJD2C, and acetyltransferase steroid receptor coactivator-1 (SRC-1) by stimulating H3K9 demethylation and acetylation (Marmorstein and Trievel, 2009). Interestingly, targeting SUV39H1, JMJD2C, and SRC-1 ameliorated obesity-related endothelia dysfunction in mice (Costantino et al., 2019). Moreover, depletion of p66Shc restored insulin response through the IRS-1/Akt/eNOS and NF-kB pathways (Natalicchio et al., 2011).

H3K27 methylation is a repressive epigenetic mark. In a type 2 diabetic mouse model, the H3K27me3 level at *RAGE* and *PAI-1* promoters was decreased relative to db/+ mice (Komers et al., 2013). As illustrated by another OVE26 mice and STZ-induced rat type 1 diabetic models, the H3K27me3 levels were decreased, and consequently the expression of Cox2 and MCP-1 in mice was enhanced (Komers et al., 2013). The proliferation of pancreatic β cells is important in adapting to the increased insulin requirement as cell proliferation weakens after birth (Dhawan et al., 2009). The level of H3K27 declined at Ink4a/Arf and HMT, EZH2, which was consistent with the elevated expression of Ink4a/Arf in older mice (Massenet et al., 2021).

Among histone methylations, the methylation of histone H4 was initially identified about half a century ago; however, its catalyzing enzymes was not clear until recently. H4K20me1/2 methylation, which is involved in gene repression, is responsible for DNA replication and damage repair (Jørgensen et al., 2013). The methylation of H4K20me1 is mediated by SEY8, whereas H4K20me2/3 is primarily mediated by SUV4-20H1 and SUV4-20H2 (Eid et al., 2016). Glucose dysregulation induced the downregulation of antioxidant gene mitochondrial superoxide dismutase (SOD), and the increased level of H4K20me3 via increased recruitment of SUV4-20H2 to SOD gene promoter induced its expression (Reddy et al., 2012). The multigenerational effects of HFD in the first two generations promoted the elevated methylation of H4K20 at the leptin promoter, which was consistent with the increased expression and serum level of leptin at 12 and 24 weeks of age in white adipose tissues (Macchia et al., 2021). Interestingly, the level of H4K20me1 was also increased in the offspring of both parents upon HFD treatment (Safi-Stibler and Gabory, 2020). These observations indicated that histone modification marks can be acquired from *in utero* HFD induction or from both parents.

### HMTs, HDMs, Writers, and Erasers in Type 2 Diabetes

Histone methylation is relatively stable and modulated by HMTs (as methylation writers) and HDMs (as methylation erasers), which cause complexity in the pathogeny of diabetes and related diseases. Typically, H3K4me is catalyzed by several HMTs, including SET1/COMPASS, mixed lineage leukemia 1–4 (MLL1-4), SET and MYND domain 2/3 (SMYD2/3), and SET7/9 (Gu and Lee, 2013). H3K9me can be modified by suppressor of variegation 3–9 homolog 1/2 (SUV39H1/2), G9a, G9a-like protein (GLP), SET domain, bifurcated 1/ERG-associated protein with SET domain (SETDB1/ESET), and Eu-HMTase1 (Table 2) (Venkatesh and Workman, 2013). In addition, H3K27me is mediated by EZH2, H3K36me by SET2, and H3K79 by Dot1 (Kim et al., 2017). These enzymes can modify these sites to different degrees to modulate the expression of downstream genes. Histone methylation can also be affected by different metabolites as cofactors or cosubstrates (Chen et al., 2020). HMTs transfer methylation groups dependent on S-adenosyl methionine (SAM), whereas JmjC domain-containing demethylases remove methyl groups dependent on α-ketoglutarate (αKG) (Matilainen et al., 2017). As a critical metabolite in the TCA cycle, αKG serves as a substrate in several anabolic processes (Noe and Mitchell, 2019). The αKG level is affected by the cell’s metabolic state and correlated to the fasting state in hepatocytes (Sivanand and Vander Heiden, 2020). Therefore, the metabolic status is associated with the fluctuation of αKG, thereby affecting the demethylating activity of the JmjC domain-containing family.

**TABLE 2:** HMT and HDM specificity, and their roles in diabetes-related phenotypes.

Classifcation	Family	Name	Specificity	Roles in diabetes-related phenotypes
HMT	PRMTs	PRMT1	H4R3	Impaired PRMT1 activity stimulated by hyperglycemia
		PRMT4	H3R2, R17, R26	Increased in diabetic models. PRMT4 inhibition suppresses the expression of insulin
		PRMT5	H2A, H4 (non-histone proteins)	
		PRMT7	H3R2	
	SET	EZH2	H3K27	Modulate β-cell dedifferentiation and cell proliferation
		NSD1-3, SETD2, SMYD2	H3K36	
		SUV39H1, SUV39H2	H3K9	Overexpression of SUV39H1 ameliorate the diabetic phenotypes
		G9a	H3K9, H3K27	Modulated insulin signaling pathway
		SET7/9	H3K4	Essential for the glucose-stimulated insulin secretion
		SET8	H4K20	
		SUV4-20H1, SUV4-20H2	H4K20	
	Seven-β-strand (7BS)	Dot1/DOT1L	H3K79	
HDMs	KDM1	KDM1A (LSD1)	H3K4me1/2, H3K9me1/2	Promotion of beige adipocyte
		KDM1B (LSD2)	H3K4me1/2	
	JMJC	KDM5A/B/C/D	H3K4me2/3	
		KDM2B (JHDM1B)	H3K36me2/1, H3K4me3	
		KDM2A (JHDM1A)	H3K36me2/1	
		KDM3A (JHDM2A, JMJD1A)	H3K9me2/1	Promotion of beige adipocyte. Depletion of JMJD1A is relevant with obesity
		KDM4A (JHDM3A, JMJD2A)	H3K9me3/2, H3K36me3/2	Enhanced in db/db mice
		KDM5A (JARID1A)	H3K4me3/2	
		KDM6B (JMJD3)	H3K27me2/3	Promotion of beige adipocyte
		PHF2	H3K9me2	Overexpression of PHF2 in mice led to improved glucose intolerance and insulin resistance

**FIGURE 3:**
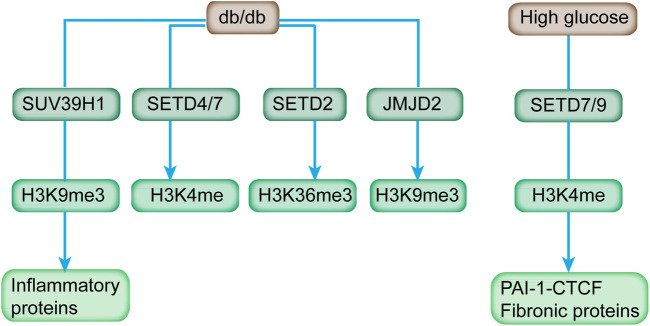
HMTs, HDMs, and respective histone methylation mark affected by diabetes and high glucose. Diabetic mice (db/db) exhibited increased expression of H3K4 methyltransferases (SETD4 and SETD7), H3K36 methyltransferase (SETD2), H3K9 demethylases (JMJD2 family), and reduced level of H3K9 methyltransferase (SUV39H1). In addition, SETD7/9 is upregulated in response to high glucose, and it modulates key fibrotic genes.

**FIGURE 4:**
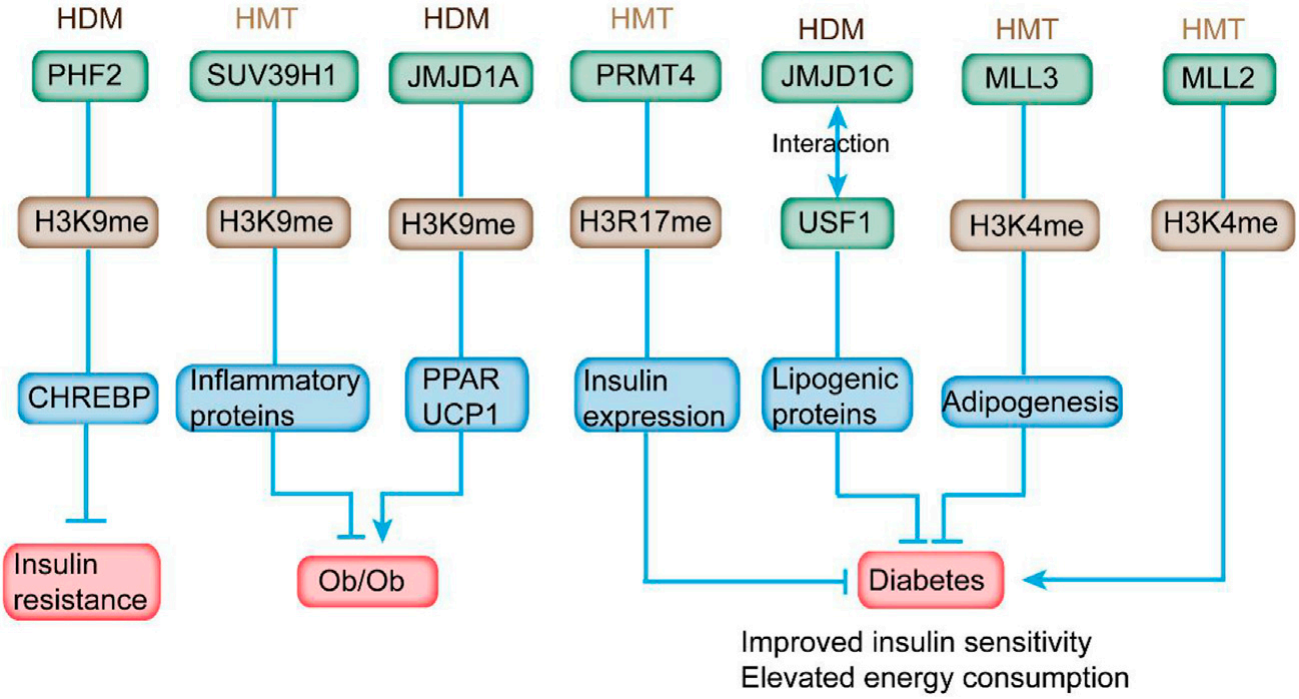
HMTs and HDMs modulate insulin sensitivity and diabetes-related metabolic syndrome through altering histone methylation status on the promoter of the respective genes. In detail, overexpression of PHF2 in mice leads to improved glucose intolerance and IR. Overexpression of SUV39H1 partially ameliorates the diabetic phenotypes. JMJD1A depletion is relevant to obesity. PRMT4 inhibition reduces the expression and secretion of insulin. Deletion of JMJD1C provides protection from high-carbohydrate diet-induced hepatosteatosis and IR. MLL3 mutation exhibits overall beneficial metabolic profile. In addition, MLL2 mutation causes insulin resistance and glucose intolerance.

SUV39H1 can mediate the methylation of H3K9me3, and its suppression is directly associated with the reduced H3K9me3 level at the promoter of inflammatory genes to affect their expression in VSMC of db/db mice (Figure 3; Table 2) (Villeneuve et al., 2010). In addition, the overexpression of SUV39H1 in db/db VSMC could partially ameliorate the diabetic phenotypes (Figure 4) (Czvitkovich et al., 2001).

SET7/9 recruitment and H3K4 methylation are the features of activated insulin genes in cells involved in insulin production, such as β cells, non-β cells, and embryonic stem cells (Batista and Helguero, 2018). The SET7/9 knockdown in monocytes reduced the H3K4me1 level at the promoter of *MCP-1* and *TNF-*α and subsequently reduced occupancies of the NF-κB subunit on promoters, indicating the dependence of NF-κB in inhibiting TNF-α-induced expression of key inflammatory genes (Sun et al., 2014). Meanwhile, transient stimulation of high glucose induced increased recruitment of SET7/9 and LSD1 to the *p65* promoter in aortic endothelial cells (Reddy et al., 2008). In rat renal mesangial cells, SET7/9 was upregulated and actively recruited to the promoter of key fibrotic genes (i.e., *PAI-1* and *CTCF*) in response to high glucose (Figure 3), accompanied with increased H3K4 occupancy (Keating and El-Osta, 2013). In addition, deficiency of *SET7/9* in the islets contributed to impaired glucose-induced Ca^2+^ mobilization and insulin secretion (Table 2). Therefore, SET7/9 is essential for glucose-stimulated insulin secretion in beta cells via modulating the euchromatin structure at the promoter of related genes through mediating histone methylation (Ogihara et al., 2009).

Previous studies reveal the association between H3K4 methylation and adipogenesis (Ge, 2012). MLL3 Δ/Δ mice harboring an H3K4 HMTs inactive mutant exhibited a significant decline of white adipose tissue accompanied with overall beneficial metabolic profile, including improved insulin sensitivity and elevated energy consumption (Figure 4) (Lee et al., 2009). These data indicate the potential application of HMT activities as targets for antidiabetic and/or anti-steatohepatitis therapeutics in certain tissues.

Haploinsufficiency in MLL2, an HMT of H3K4, induced hyperglycemia and hyperinsulinemia, which contributed to hepatic fat deposition and irregular plasma triglyceride in mice (Goldsworthy et al., 2013). In addition, MLL2 mutation caused IR and glucose intolerance in mice (Figure 4). Considering that HMT is responsible for the addition of H3K9me1/2, G9a was downregulated in diabetic mouse model. However, G9a could affect insulin sensitivity independent of methyltransferase activity. It modulated the insulin signaling pathway *via* mediating the structure of the transcriptional factor HMGA1, a key regulator in insulin reporter (Xue et al., 2018). In addition, G9a restoration in db/db mice improved IR and mitigated hyperglycemia and hyperinsulinemia (Chiefari et al., 2018). As a critical HMT for H3K27me3, EZH2 modulated β-cell dedifferentiation and proliferation during pancreatic endocrine specification (Dahlby et al., 2020). Homozygous EZH2-specific depletion promoted mild glucose intolerance and decreased β-cell mass (Arnes and Sussel, 2015). In addition, a reduced level of H3K27me3 was observed in β cells from human T2DM donors (Lu et al., 2018). Another HMT, PRMT4, which was responsible for H3R17 methylation, was increased in the retinal pigment epithelial layer of diabetic rats to promote cell death (Wang et al., 2017). High-glucose stimulation induced the expression of PRMT4 in INS-1 and HIT-T15 pancreatic β cells (Kim et al., 2015). PRMT4 inhibition by drugs or knockdown inhibited the expression of insulin and secretion stimulated by high glucose in primary pancreatic islets. Furthermore, overexpression of defective PRMT4 reduced the level of insulin expression (Figure 4) (Kim et al., 2015). Goto-Kakizaki rats exhibited enzymatic impaired PRMT1 activity and defective protein methylation when stimulated by postprandial hyperglycemia, indicating the potential involvement of protein methylation in mediating insulin secretion (Iwasaki, 2009).

At present, the multitude of demethylases has been identified with specific catalyzing activity toward different histone methylations (Bhaumik et al., 2007). Histone methylation is dynamic, and it potentially affects multiple diseases, including diabetes (Gottlieb et al., 2010). As depicted by a qPCR array screen, db/db mice exhibited increased expression of H3K4 methyltransferases (SETD4 and SETD7), H3K36 methyltransferase (SETD2), and H3K9 demethylases (JMJD2 family), which can be relieved by Losartan, an Ang II type 1-receptor blocker (Figure 3) (Reddy et al., 2014).

JMJD1A is responsible for demethylating mono- and di-methyl H3K9 at the promoters to gain access for gene transcription (Marmorstein and Trievel, 2009). Its depletion is relevant with obesity, reduced level of genes related to active metabolism (such as peroxisome proliferator-activated receptor and medium-chain acyl-CoA dehydrogenase) in skeletal muscle, and declined expression of cold-induced uncoupling protein 1 in brown adipose tissues of rodents (Figure 4) (Inagaki, 2018). JMJD1A also modulates genes involved in energy homeostasis, including anti-adipogenesis (*Nr2f2* and *GATA2*), regulation of fat storage (*Apoc1*), glucose transport (Slc2a4), and predisposing genes of T2DM (*ADAMTS9*) in white adipose tissue (WAT) (Lafortuna et al., 2017). JMJD1A^−/−^ mice displayed hypothermia induced by fasting and increased respiratory quotient (Figure 4) (Tateishi et al., 2009; Lafortuna et al., 2017). Therefore, JMJD1A plays an important role in modulating energy expenditure and fat deposition, indicating its hitherto uncharacterized role in metabolic syndrome.

Based on previous reports, several HDMs had the potential to interact with transcriptional factors. For example, plant homeodomain finger protein 2 (PHF2), a type of H3K9 HDM, is essential for the transcriptional activation of ChREBP, which is a critical regulator in lipid and glycolytic metabolism (Kim et al., 2014). CHREBP plays an important role in strengthening lipogenesis under IR condition (Benhamed et al., 2012). Overexpression of PHF2 in mice led to improved glucose intolerance and IR and reduced expression of proinflammatory genes (Figure 4) (Okuno et al., 2013). PHF2 can promote SCD1 activity and elevate the ratio of monounsaturated fatty acids to saturated fatty acid, providing protection from lipotoxicity, oxidative stress, and IR (Bricambert et al., 2018). Liver-specific deletion of a HDM, JMJD1C, could provide protection from high-carbohydrate diet-induced hepatosteatosis and IR and contribute to the decreased expression of lipogenic genes through its interaction with USF1 (Figure 4) (Viscarra and Sul, 2020). Single-nucleotide polymorphism in JMJD1C is connected to the progression of T2DM, indicating the potential involvement of JMJD1C in the development of metabolic diseases as evaluated by genome-wide association studies (Kim et al., 2018). These studies have indicated the potential effects of HMTs and HDMs on the pathogenesis of T2DM.

### Epigenetic-Based Therapies for Type 2 Diabetes

Collectively, we discussed that histone methylations were casually involved in the expression of genes implicated in the pathogenesis of T2DM (Sun et al., 2015). Recent studies suggested that targeting histone methylation might reverse the deleterious epigenetic marks and modulate the expression levels of genes related to T2DM. Multitude drugs and chemicals targeting histone methylations have been proposed for the treatment of this disease. More recently, inhibitors that target chromatin-associated epigenomic writers such as EZH2, erasers such as LSD1, and readers such as BRD4 are being considered as therapeutic agents (Rosen et al., 2018). The use of BET/BRD inhibitor JQ1 was highlighted in the treatment of cancer. However, it may not be suitable for the treatment of diabetes due to severe side effects, broad activity on multiple pathways, and lack of cell-type specificity.

**FIGURE 5:**
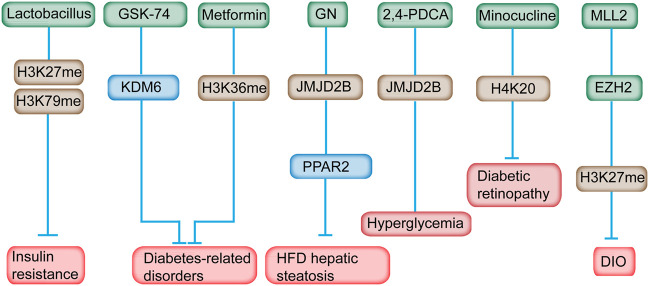
Inhibitors of histone methylation and their targets for the prevention of diabetes. For example, *Lactobacillus* can modulate H3K27 and H3K79 methylation in IR. Inhibition of KDM6A with GSK-J4 reduces Cry1 expression and sensitizes leptin signaling to combat obesity‐related diseases. Metformin reversed the H3K36me mark in prediabetic and diet-induced obesity mouse model. GN mediated HFD-induced hepatic steatosis by reducing the JMJD2B and PPARγ2 levels. Minocycline reversed diabetes-related chronic inflammation by modifying the methylation state of H4K20me. In addition, GSK126, an EZH2-specific inhibitor, alleviates the obesity phenotype in diet-induced obese mice.

*Lactobacillus* supplementation can modulate the histone methylation profile in IR (Sharma et al., 2020). H3K27 and H3K79 methylation state can be modulated by *Lactobacillus* supplementation (Figure 5) (Sharma et al., 2020). *Lactobacillus* supplementation predominantly prevented methylation and demethylation of H3K79me2 and H3K27me3, respectively, making it not an ideal drug for histone methylation-related diseases (Sharma et al., 2020). H3K27 methylation is associated with transcription repression, whereas H3K79 is correlated with transcriptional activation (Lin et al., 2010). The correlation of H3K37me3 in metabolic disorders has been identified (Wei et al., 2021). H3K27me3 can be removed by KDM6. Pharmacological inhibition of KDM6 members with GSK-J4 relieved the progression of nephropathy in diabetic db/db mice (Figure 5) (Chen et al., 2021). Inhibition of KDM6A reduces Cry1 expression and sensitizes leptin signaling to combat obesity‐related diseases (Wei et al., 2021). Therefore, GSK-J4, a KDM6A inhibitor, could serve as an attractive drug for obesity and metabolic disorders.

GSK126, an EZH2-specific inhibitor, alleviates the obesity phenotype by promoting the differentiation of thermogenic beige adipocytes in diet-induced obese mice (Figure 5) (Wu et al., 2018). After GSK126 administration, the H3K27me3 and mRNA levels of the key transcription factors in adipocytes differentiation increased significantly, however, the number of lipid droplets and lipid content in the GSK126 group didn’t change much, possibly owning to some unknown side effects or drug toxicity of GSK126 in physiological functions of cells (Wu et al., 2021). An MLL1 small-molecule inhibitor, MI-2, administered at the appropriate time postinjury, may be an ideal therapeutic drug to decrease chronic inflammation of wounds among patients with diabetes (Kimball et al., 2017).

Metformin supplementation has long been implicated in the treatment of T2DM. Chromatin modification alteration is induced by metformin at the enhancer element of the *ATM* gene, a locus that is, relevant with metformin response in primary human hepatocytes (Bridgeman et al., 2018). Metformin reversed the H3K36me2 mark in prediabetic and diet-induced obesity mouse model (Figure 5) (Nie et al., 2017). In addition, metformin was found directly targeted the H3K27me3 demethylase KDM6A/UTX (Cuyàs et al., 2018). Metformin was revealed to modulate the level of multiple histone methyltransferases, the activity of SIRT1 and the effects of DNMT inhibitors (Bridgeman et al., 2018). Consequently, these alterations might influence the epigenome and gene expression, and subsequently leading to the antidiabetic properties of metformin. As mentioned above, the effect of metformine can alter histone methylation and gene expression which leaves much uncertainty related to the overall effect of metformin on the epigenome, on gene expression, and on the subsequent effect on the health of metformin users.

Gomisin N (GN) improves hepatic steatosis induced by HFD (Jung et al., 2017). In-depth study revealed that GN effectively reduced the expression level of JMJD2B and subsequently decreased the expression level of PPARγ2 and downstream genes, indicating the potential mechanism in GN-mediated HFD-induced hepatic steatosis (Figure 5) (Jang et al., 2017). JMJD2A inhibition induced by a chemical inhibitor, namely, 2,4-PDCA, suppressed VSMC migration, proliferation, and inflammation accompanied by decreased H3K9me3 caused by hyperglycemia *in vitro* and mitigated neointimal formation in balloon-injured diabetic rats (Qi et al., 2015).

LSD1 plays a central epigenetic role in various metabolic disorders, including obesity-associated diseases, neurological disorders, and cancer (Cuyàs et al., 2019). Accordingly, extra virgin olive oil contained a naturally occurring phenolic inhibitor of LSD1 and potentially exerted a beneficial effect on the treatment of obesity-associated diseases (Cuyàs et al., 2019).

Hepatic insulin sensitivity can also be modified by specific dietary components that are involved in epigenetic modifications (Maude et al., 2021). In mammals, SAM can provide methyl groups for DNA, RNA, and histones (Ouyang et al., 2020). The content of SAM is determined by the intake of its precursor, methionine and co-factor folate (vitamin B9), betaine, and vitamins B2, B6, and B12. Previous studies observed that the serum level of methyl-related metabolites correlated with IR (Zhou et al., 2011). Apart from being a methyl donor, folate is also related to LSD1 (Chen et al., 2006).

Minocycline, a tetracycline antibiotic, can reverse the diabetic-related chronic inflammation in the retina of rodents (Wang et al., 2017). Meanwhile, the methylation state of H4K20me1/2, which is associated with DNA damage response, was increased in the retina of diabetic rats, and minocycline treatment restored the methylation status of H4K20 (Wang et al., 2017). Therefore, the favorable effect of minocycline on the development of diabetic retinopathy is partially attributed to its effect on the changed histone methylation.

Apart from its therapeutic potential, epigenetic modification could also serve as disease biomarkers, which promotes early detection of disease progression and improves the estimates of future disease risk (Hornschuh et al., 2021). A total of 63 genomic regions associated with insulin sensitivity are identified from neonatal blood spot samples (Maude et al., 2021). Such genomic regions may facilitate appropriate lifestyle interventions as biomarkers to reduce IR-related diseases. Recently, Sadeh et al. utilized ChIP-seq to identify liver-specific histone modification marks in plasma-free nucleosomes (Sadeh et al., 2021). In spite of the relatively small sample size and patient heterogeneity, histone modification enrichment in liver diseases can be detected, and it can facilitate the high-throughput interrogation of disease signature in patient blood samples.

## Conclusion

The interaction between genetics and epigenetics plays an important role in the pathogenesis of T2DM. Epigenetics refers to the phenomenon of intergenerational inheritance through cell division without changing the base sequence of DNA genetic material. Among epigenetics, histone methylation is the most important modification mode, which serves as a complicated link between genotype and phenotype. A number of studies have shown that methylation modification can affect the development of pancreatic β cells, insulin sensitivity, and secretion; thus, it is considered as a possible mechanism of T2DM. Designing therapeutic targets for epigenetic inheritance, particularly histone methylation, may provide a new approach for the treatment of T2DM.

Recent studies have also revealed that environmental factors such as energy metabolism disorders can lead to epigenetic alterations, resulting in “metabolic memory,” affecting the development and secretion of islet β cells, reducing body’s sensitivity to insulin, and ultimately leading to the occurrence of T2DM. These epigenetic changes can be corrected and reversed by special diets, drugs, and lifestyle remodeling, providing new ideas for the prevention of T2DM and developing potential drug targets for treatment.

Histone modification can be catalyzed by HMTs (writers) and removed by HDMs (erasers). Recent studies have emphasized the importance of histone methylation alteration in controlling adipogenesis and energy homeostasis. The causal relationship between diet-induced obesity and histone methylation regulated genes associated with nutrition balance, which required further investigation. Meanwhile, the utilization of genome-wide technologies in gene expression and genetic variation in patients with T2DM have provided new candidate genes. However, the association between epigenetic modification alterations and T2DM remains unclear. Thus, analyzing the role of histone modification and DNA methylation in the pathogenesis of T2DM and uncovering its complications are challenging.

Some of the epigenetic changes associated with obesity affect genes known to increase the risk of diabetes. Other epigenetic changes affect genes not specifically related to the disease, but they play a role in metabolism. A previous study identified a series of genes that had not previously been shown to play a role in diabetes. In further tests, they found that at least some of these genes did indeed regulate insulin’s effect on sugar absorption (Multhaup et al., 2015); This provides insights into new potential therapeutic targets for T2DM. In addition to providing clues for drug development, new epigenetic tests should be developed to determine who will develop diabetes earlier, thus offering more hope for prevention of the disease. In addition, the understanding of the mechanism of gene transcription at the epigenetic level will help us to further study the prevention and control of diabetes and its complications and provide new ideas for treatment.
